# Electrochemical studies of a high voltage Na_4_Co_3_(PO_4_)_2_P_2_O_7_–MWCNT composite through a selected stable electrolyte†

**DOI:** 10.1039/d0ra02349c

**Published:** 2020-04-22

**Authors:** P. Ramesh Kumar, R. Essehli, H. B. Yahia, R. Amin, I. Belharouak

**Affiliations:** Qatar Environment and Energy Research Institute (QEERI), Hamad Bin Khalifa University, Qatar Foundation 34110 Doha Qatar; Energy and Transportation Science Division, Oak Ridge National Laboratory Oak Ridge TN USA aminr@ornl.gov

## Abstract

Cathode materials that operate at high voltages are required to realize the commercialization of high-energy-density sodium-ion batteries. In this study, we prepared different composites of sodium cobalt mixed-phosphate with multiwalled carbon nanotubes (Na_4_Co_3_(PO_4_)_2_P_2_O_7_–MWCNTs) by the sol–gel synthesis technique. The crystal structure and microstructure were characterized by using PXRD, TGA, Raman spectroscopy, SEM and TEM. The electrochemical properties of the Na_4_Co_3_(PO_4_)_2_P_2_O_7_–20 wt% MWCNT composite were explored using two different electrolytes. The composite electrode exhibited excellent cyclability and rate capabilities with the electrolyte composed of 1 M sodium hexafluorophosphate in ethylene carbonate:dimethyl carbonate (EC:DMC). The composite electrode delivered stable discharge capacities of 80 mA h g^−1^ and 78 mA h g^−1^ at room and elevated (55 °C) temperatures, respectively. The average discharge voltage was around 4.45 V *versus* Na^+^/Na, which corresponded to the Co^2+/3+^ redox couple. The feasibility of the Na_4_Co_3_(PO_4_)_2_P_2_O_7_ cathode for sodium-ion batteries has been confirmed in real time using a full cell configuration *vs.* NaTi_2_(PO_4_)_3_–20 wt% MWCNT, and it delivers an initial discharge capacity of 78 mA h g^−1^ at 0.2C rate.

## Abstract 2

Na_4_Co_3_(PO_4_)_2_P_2_O_7_–MWCNT composites in 1 M NaPF_6_ in EC:DMC electrolytes deliver stable discharge capacities of 80 mA h g^−1^ and 78 mA h g^−1^ at normal and elevated temperatures, respectively. In a full cell configuration *vs.* NaTi_2_(PO_4_)_3_–MWCNT, they deliver an initial discharge capacity of 78 mA h g^−1^ at 0.2C rate.

## Graphical abstract

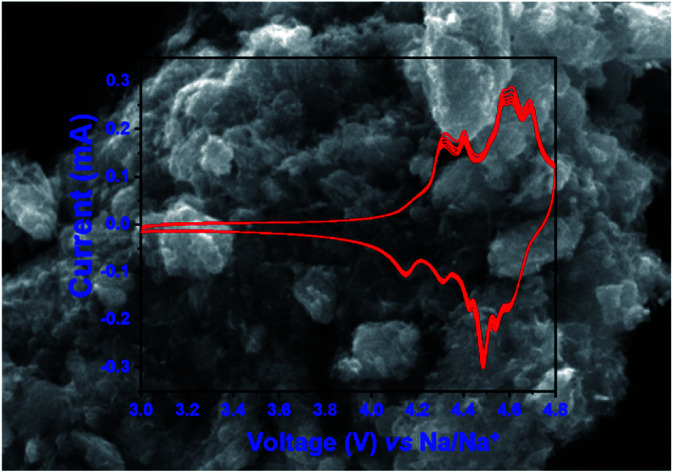

## 1. Introduction

Sodium-ion batteries (SIBs) are an alternative for the well-established lithium-ion batteries because of the low cost of sodium and its wide availability besides similar electrochemistry to lithium.^1–3^ However, limitations like low energy density, poor cyclability, and slow sodium kinetics prevent SIBs from achieving their commercialization potential.^4,5^ To overcome these challenges, research on high-voltage and high-capacity cathode materials for SIBs is being conducted.^6^ Different types of cathode materials have been studied, including the layered oxides such as Na_0.44_MnO_2_,^7^ NaCoO_2_,^8^ O3-NaNi_0.5_Mn_0.5_O_2_,^9^ and P2-Na_2/3_Fe_1/2_Mn_1/2_O_2_;^10^ phosphates such as NaFePO_4_,^11^ Na_1.8_Fe_3_(PO_4_)_3_, Na_2_M_2_Fe(PO_4_)_3_ (where M = Ni and Co), Na_4_MnV(PO_4_)_3_, Na_3_V_2_(PO_4_)_3_,^12^ NaTi_2_(PO_4_)_3_,^13^ Na_3_V_2_(PO_4_)_3_F_3_,^14^ Na_3_V_2_O_2_(PO_4_)_3_F,^15^ and Na_2_FeP_2_O_7_;^16^ sulfates such as NaFe(SO_4_)_2_ (ref. 17) and NaFe_2_(PO_4_)(SO_4_)_2_;^18^ and the Prussian blue derivatives Na_2_NiFe(CN)_6_ (ref. 19 and 20) and Na_1.89_Mn[Fe(CN)_6_]_0.97_ (ref. 21 and 22) Recently, new mixed-polyanion compounds including NaM_2_(PO_4_)(SO_4_)_2_, Na_4_M_3_(PO_4_)_2_(P_2_O_7_) and Na_3_MPO_4_CO_3_ [M = transition metal] have been suggested as cathode materials for SIBs owing to their chemical stability, high operating cell voltage^4,23^ and ease of processing. In 2001, Sanz *et al.* reported the synthesis and characterization of Na_4_M_3_(PO_4_)_2_(P_2_O_7_) [M = Mn, Co and Ni] compounds, including activation energies and ionic conductivity studies.^24,25^ Kim *et al.* studied the electrochemical mechanism of Na_4_Fe_3_(PO_4_)_2_(P_2_O_7_) in SIBs. Na_4_Fe_3_(PO_4_)_2_P_2_O_7_ exhibited a voltage plateau at 3.2 V *vs.* Na^+^/Na.^26,27^ Zhang *et al.* presented the preliminary electrochemical results of Na_4_Ni_3_(PO_4_)_2_P_2_O_7_ with ionic liquid electrolytes and the average voltage was 4.8 V *vs.* Na^+^/Na.^28^ Nose *et al.* explored the electrochemical properties of pure Na_4_Co_3_(PO_4_)_2_P_2_O_7_ and Mn and Ni co-doped Na_4_Co_3_(PO_4_)_2_P_2_O_7_. Despite having a high current density of 850 mA g^−1^, the rate capabilities of these SIB cathode materials are limited due to poor electronic conductivities.^29,30^ Recently, Tang *et al.* reported highly conductive CNT-decorated Na_4_Mn_2_Co(PO_4_)_2_P_2_O_7_ microspheres for high-voltage SIBs, which delivered an initial discharge capacity of 93.5 mA h g^−1^, and the capacity retention was around 77% after 100 cycles at 0.5C rate. Also, they succeeded in obtaining a high stable capacity of 99.5 mA h g^−1^ at 0.5C rate after substitution with Al^3+^ in the Co^2+^ site of the Na_4_Co_3_(PO_4_)_2_P_2_O_7_ structure, which could improve the ionic conductivity as well as structural stability at a high voltage.^31,32^

In this work, we have investigated the electrochemical stability and cyclability of the high-voltage cathode Na_4_Co_3_(PO_4_)_2_P_2_O_7_ (NCPP) with 20 wt% of multiwalled carbon nanotubes (MWCNTs), abbreviated as NCPP–CNT from here on, using suitable high voltage electrolytes. The NCPP–CNT composites were prepared by mixing simple sol–gel synthesized bare Na_4_Co_3_(PO_4_)_2_P_2_O_7_ with MWCNTs, followed by heat treatment under an argon atmosphere. Structural and microstructural characterizations of the prepared composites were performed by using PXRD, TGA, Raman spectroscopy, SEM, and TEM-EDX. Additionally, two different electrolytes were prepared, and their electrochemical stability window was found using linear sweep voltammetry (LSV). Furthermore, the full-cell electrochemical performance of NCPP–CNT//NaTi_2_(PO_4_)_3_–MWCNT at 0.2C rate was studied using a stable electrolyte comprising 1 M NaPF_6_ in EC:DMC. The obtained results provide insights into the materials for further improvement toward commercialization.

## 2. Experimental

### 2.1. Synthesis of Na_4_Co_3_(PO_4_)_2_P_2_O_7_–carbon nanotube composites

NCPP was prepared using a sol–gel method by taking a stoichiometric mixture of Co(CH_3_COO)·4H_2_O, (NH_4_)_2_HPO_4_ (Merck, ≥99%), and Na_4_P_2_O_7_ (Sigma-Aldrich, 99.99%). The three compounds were dissolved separately in DI water forming the solutions I, II, and III. The solutions II and III were mixed together and then added dropwise to solution I under continuous stirring. Then, the resulting solution was slowly evaporated to dryness by constant stirring at 80 °C. The residual precursor was ground in an agate mortar and heat-treated in air in an alumina crucible at 400 °C for 24 h and at 650 °C for 24 h to obtain bare NCPP. To improve the electronic conductivity of the bare NCPP sample, it was mixed with different amounts of MWCNTs to prepare NCPP–CNT composites. These samples were resintered at 650 °C for 6 h under an argon atmosphere. Since the composite NCPP–20 wt% CNT showed better electrochemical performance compared to NCPP–10 wt% CNT and NCPP–5 wt% CNT composites, for further characterizations and electrochemical studies, only this composite electrode was studied.

NaTi_2_(PO_4_)_3_–MWCNT composite anode material was prepared by a single-step solid–state reaction, as reported earlier.^33^ Initially, stoichiometric amounts of nano-sized TiO_2_, NaH_2_PO_4_ and NH_4_H_2_PO_4_ were ball-milled for 6 h at 350 rpm. Later, 10 wt% of MWCNTs were added to the precursor mixture and further ball-milled for 1 h to obtain a uniform mix. The precursor mixture was further calcined at 900 °C for 10 h under a nitrogen atmosphere to obtain a pure crystalline NaTi_2_(PO_4_)_3_–MWCNT composite.

### 2.2. Characterization

The purity of NCPP–CNT composites was analysed using PXRD. The PXRD data were collected at room temperature over the 2*θ* range of 5°–80° with a step size of 0.01° using a Bruker D8 advanced diffractometer operating with CuK_α_ radiation.^34^ The particle size, morphology and uniformity were characterized using a field emission scanning electron microscope (FE-SEM, FEI, Quanta 650, Japan). The carbon content of the NCPP–CNT samples was determined by thermogravimetric analyses, which were carried out in an oxygen atmosphere using TA instruments (TG800, USA).

The electrochemical studies of the composite electrodes were conducted in CR2032 coin cells. The composite electrodes were prepared by mixing 80 wt% of active material with 10 wt% of Super P carbon black and 10 wt% of carboxymethyl cellulose (CMC) binder. The obtained slurry was coated on a piece of Al foil and cut into 14.5 mm diameter circular electrodes. Sodium metal was used as an anode for sodium half cells. A 1 M solution of NaPF_6_ in ethylene carbonate:dimethyl carbonate (EC:DMC) and 1 M solution of NaClO_4_ in propylene carbonate with 5 v/v% of fluoroethylene carbonate (PC:5% FEC) were used as electrolytes. Coin cells were assembled in an argon-filled glove box (VAC atmosphere, USA) using borosilicate glass-fiber as a separator (Whatman GF/D). The cyclic voltammetry plots were obtained using VMP3 Biologic (SP-300, France) in the voltage window of 2.5 and 4.8 V. Furthermore, the stability of the electrolyte was determined using three-electrode cells. Three-electrode cells were assembled with a glass fiber separator (Whatman GF/D), a platinum wire as the working electrode and sodium metal as the counter and reference electrodes in an argon-filled glove box (VAC, O_2_ and H_2_O < 1 ppm). LSV measurements for the anodic stability of the above-mentioned electrolytes were obtained from −1 V to 5.5 V for 1 M NaClO_4_ in PC:5% FEC and from −1 V to 7 V for 1 M NaPF_6_ in EC:DMC *vs.* Na^+^/Na with a scan rate of 10 mV s^−1^. The Na-ion full cells were fabricated using the same separator and 1 M solution of NaPF_6_ in ethylene carbonate:dimethyl carbonate (EC:DMC) as an electrolyte with NaTi_2_(PO_4_)_3_–MWCNT as the anode. The Na half and full cells were galvanostatically cycled in the voltage windows of 2.5–4.8 V and 0.5–3.25 V, respectively, using an automatic battery cycler system (Arbin, LBT21084, USA). Electrochemical impedance spectroscopy (EIS) was performed in the frequency range of 7 MHz–50 mHz with a sinusoidal voltage amplitude of 10 mV using VMP3 Biologic, France. The obtained EIS spectra were fitted using an equivalent circuit model built in the *Z-View* software. Numerical values were extracted from the EIS data using a complex non-linear least-squares regression analysis.

## 3. Results and discussion

**Fig. 1:**
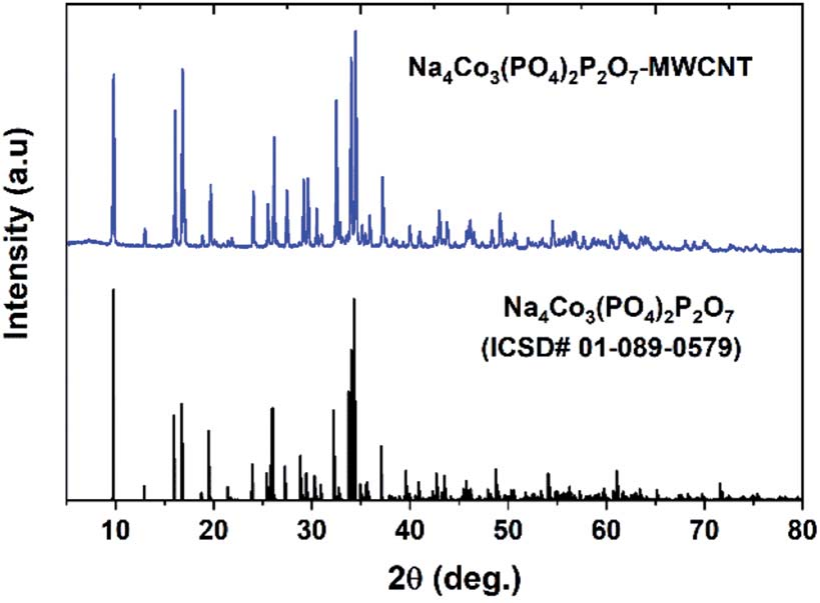
The powder XRD pattern of the Na_4_Co_3_(PO_4_)_2_P_2_O_7_–MWCNT composite along with a reference.

Fig. 1 shows the PXRD pattern of the NCPP–CNT composite along with a reference pattern (ICSD #01-089-0579). The PXRD pattern was indexed using the orthorhombic space group (*Pna*2_1_) and the obtained lattice parameters of *a* = 18.01371(3) Å, *b* = 10.51639(2) and *c* = 6.494313(1) Å and *V* = 1242.1324(1) Å^3^ were in good agreement with previous reports.^24^ The crystallite size (*D*) was calculated using the Scherrer's formula: *β* cos(*θ*) = 0.94*λ*/*D*; here, *β* is the full width-at-half-maximum of the reflection.^35^ The calculated crystallite size of the NCPP–CNT composite was approximately 74 nm.

**Fig. 2:**
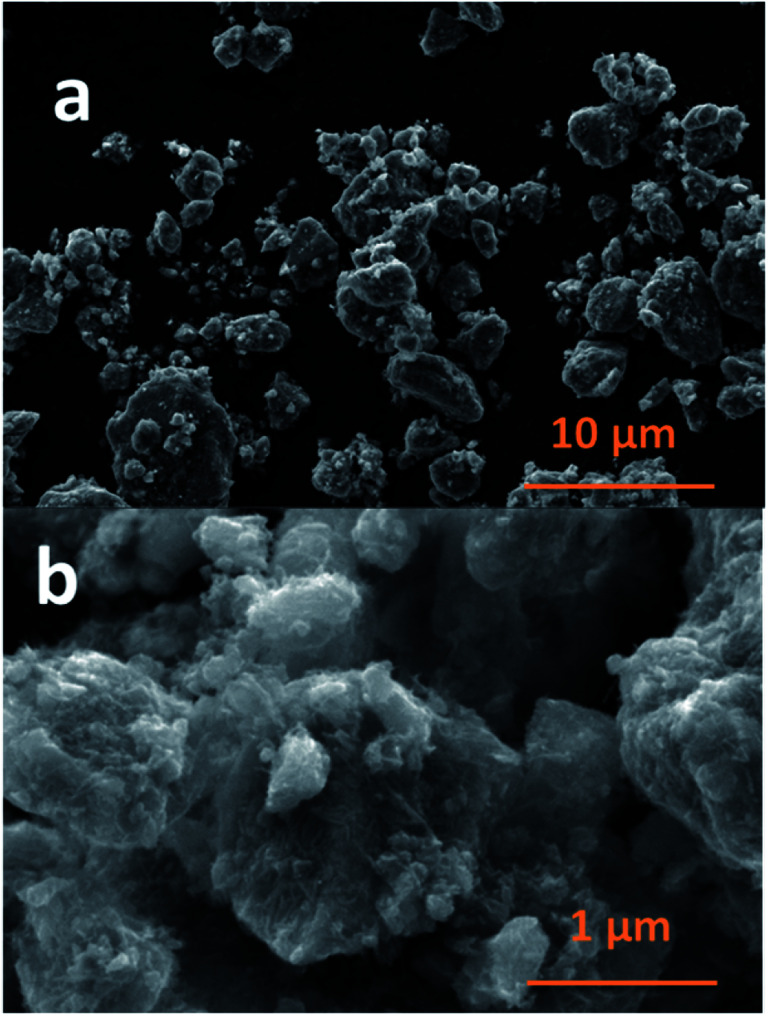
SEM images of the Na_4_Co_3_(PO_4_)_2_P_2_O_7_–MWCNT composite in different magnifications.

**Fig. 3:**
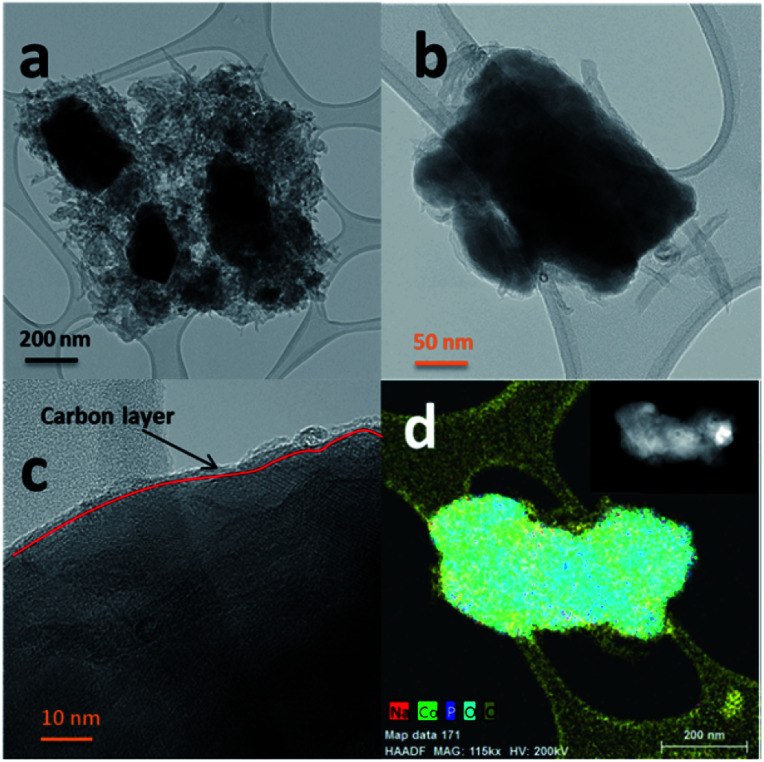
(a and b) TEM images of the Na_4_Co_3_(PO_4_)_2_P_2_O_7_–MWCNT composite in different magnifications. (c) HR-TEM image and carbon nanotube layer on the surface and (d) EDX mapping of Na_4_Co_3_(PO_4_)_2_P_2_O_7_–MWCNT composite particle.

The SEM images of NCPP–CNT at two different magnifications are presented in Fig. 2. They show a uniform distribution of carbon nanotubes in the composite electrode and the NCPP particles appear to be agglomerated. The TEM images of NCPP–CNT (Fig. 3a–c) show the CNTs wrapping around the NCPP particles. The average particle size of NCPP is around 200 nm, as seen from Fig. 2.

On the other hand, the EDX mapping confirmed the presence of sodium, cobalt, phosphorous, oxygen and carbon in the NCPP–CNT sample (Fig. 3d). The carbon content in the NCPP–CNT composite was estimated by TGA and the obtained value was 16.7% (Fig. S1†). Fig. S2† shows the Raman spectrum of the NCPP–CNT composite. The bands at 1355 and 1585 cm^−1^ are assigned to D and G bands, respectively. A strong D band corresponds to *A*_1g_ symmetry and is present in the disordered forms of carbon. A weak G band is present due to a hexagonal ring structure with *E*_2g_ symmetry of the graphitic nature of CNTs. An overtone of the D band is present at 2720 cm^−1^ and is assigned to a G′ band. Another band present is the D + G (G′′) band at 2950 cm^−1^, often referred to as a combination band, at a frequency roughly the sum of the D and G bands.^36,37^ From Fig. S2,† it can be summarized that the multiwalled carbon nanotubes in this composite are highly disordered. Physical characterizations performed by using PXRD, SEM, TEM-EDX, TGA, and Raman spectroscopy confirmed the phase purity and uniform distribution of the multiwalled carbon nanotubes in the NCPP–CNT composite.

**Fig. 4:**
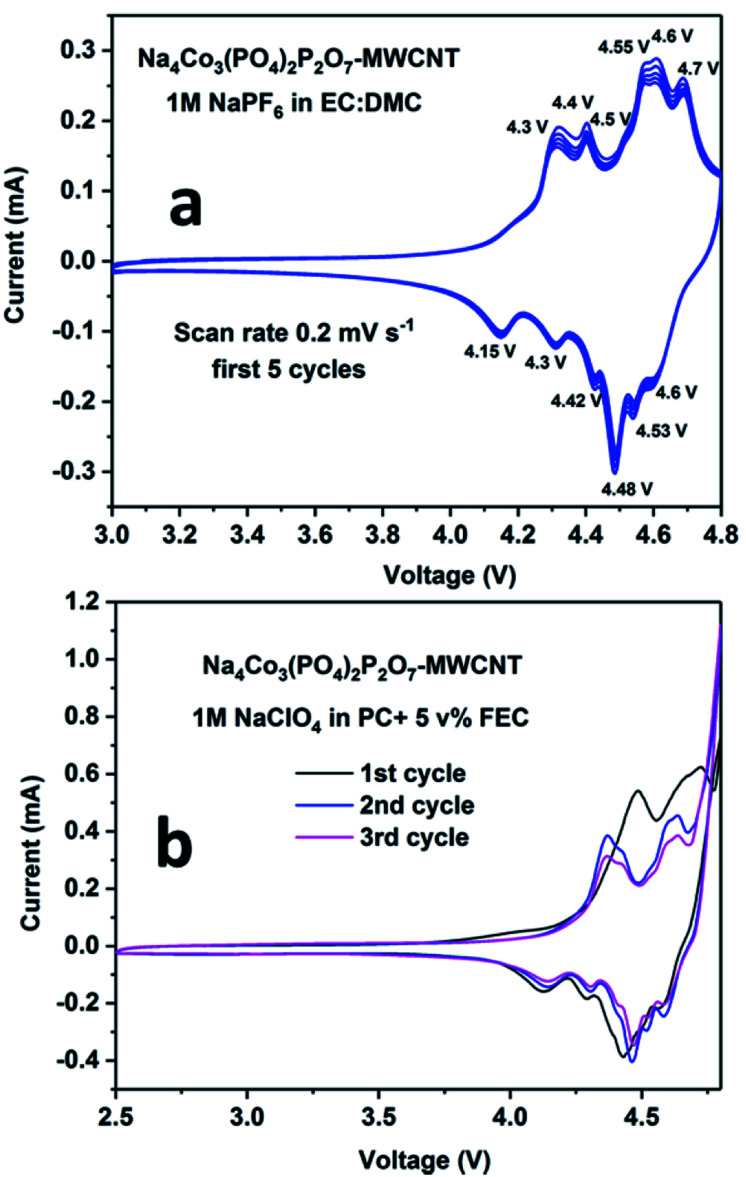
Cyclic voltammetry plots for the Na_4_Co_3_(PO_4_)_2_P_2_O_7_–MWCNT composite in (a) 1 M NaPF_6_ in EC:DMC and (b) 1 M NaClO_4_ in PC + 5% FEC electrolytes.

The cyclic voltammetry plots for the NCPP–CNT composite with two different electrolytes are shown in Fig. 4. The NCPP–CNT composite shows stable cycling in the electrolyte containing 1 M NaPF_6_ in EC:DMC when compared to that in the electrolyte containing 1 M NaClO_4_ in PC + 5% FEC. The latter electrolyte starts degrading/decomposing at around 4.7 V. From Fig. 4, it can be seen that the NCPP–CNT cyclic voltammetry plot has six small redox peaks in the voltage window from 4.3 V to 4.7 V. All these peaks are attributed to the Co^2+/3+^ redox couple in the Na_4_Co_3_(PO_4_)_2_P_2_O_7_ compound, which has four different activation sites.^38^ Due to the presence of several redox processes involved in NCPP–CNT and 1 M NaClO_4_ in PC + 5% FEC electrolyte, as shown in the cyclic voltammetry curve (Fig. 4), the details of the reaction mechanism are outside the scope of this study.

**Fig. 5:**
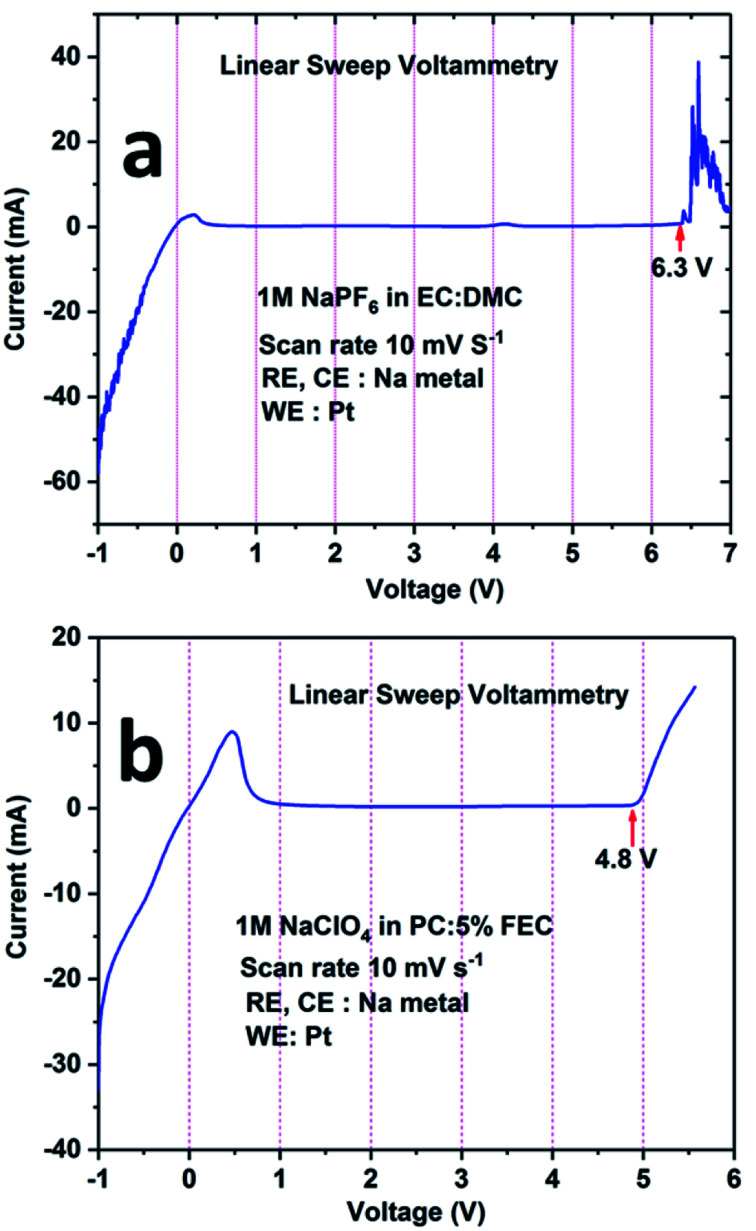
Linear sweep voltammetry curves for the electrolytes: (a) 1 M NaPF_6_ in EC:DMC and (b) 1 M NaClO_4_ in PC:5% FEC.

The electrochemical stability windows for both electrolytes (*i.e.*, 1 M NaPF_6_ in EC:DMC and 1 M NaClO_4_ in PC + 5% FEC) were determined by recording the linear sweep voltammetry curve in three-electrode set-ups. From Fig. 5a, it can be seen that the electrolyte containing 1 M NaPF_6_ in EC:DMC is electrochemically stable between 1 V and 6 V. For potentials below 1 V (the cathodic region), a current flow is observed; however, it is minor considering the small Pt wire electrode area. In the anodic region, oxidation of the electrolyte is observed above 6.3 V. Similarly, from Fig. 5b, it can be inferred that the electrolyte containing 1 M NaClO_4_ in PC + 5% FEC is electrochemically stable between 1 V and 4.8 V. For potentials below 1 V, there is an intense current peak probably related to the decomposition of FEC. On the other hand, above 4.8 V, oxidation of the electrolyte is observed. Based on these results, it was concluded that 1 M NaClO_4_ in PC + 5% FEC was less electrochemically stable as compared to 1 M NaPF_6_ in EC:DMC; therefore, the latter electrolyte was used in the study of the electrochemical performance of the NCPP–CNT composite electrode.

**Fig. 6:**
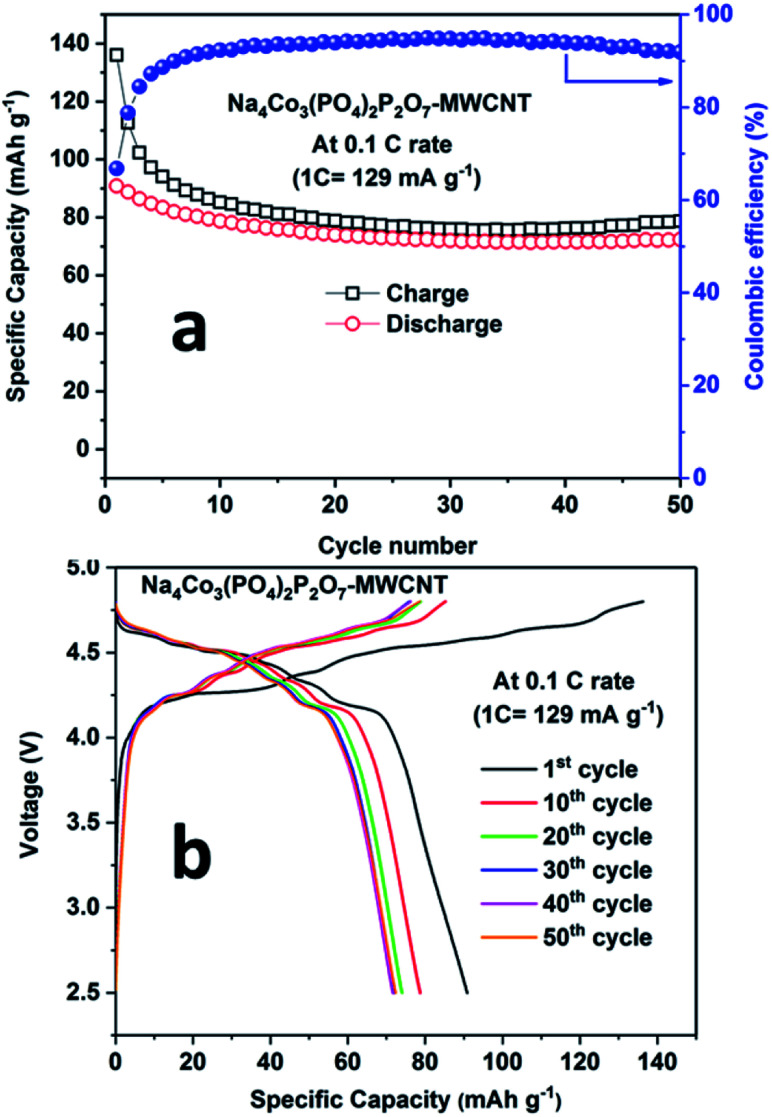
a) Cyclability results and (b) charge–discharge curves for the Na_4_Co_3_(PO_4_)_2_P_2_O_7_–MWCNT composite at 0.1C rate at room temperature.

During the first cycle, the NCPP–CNT composite electrode delivered charge and discharge capacities of around 138 mA h g^−1^ and 92 mA h g^−1^, respectively, with small six different potential plateau regimes (Fig. 6b). The irreversible capacity loss at this first cycle might be due to electrolyte decomposition and formation of an unstable SEI layer. After 5 cycles, the battery delivered a stable discharge capacity of 80 mA h g^−1^ and after 50 cycles, ∼90% of capacity was retained (Fig. 6a). From the charge–discharge curves, it can be observed that the sloping region starts above 4.6 V, and it has several small voltage plateau regions that might be correlated to the existence of four different active cobalt sites. The average voltage or midpoint voltage of the discharge curve is 4.45 V *versus* Na^+^/Na. For comparison, charge–discharge cycling of the NCPP–CNT composite electrode in 1 M NaClO_4_ in PC + 5% FEC was performed. However, endless charging was observed during the first charge, which may be due to the decomposition of the electrolyte (Fig. S3†).

In order to check the cyclability of NCPP–CNT at extreme conditions, the NCPP–CNT composite electrode was also cycled in a half-cell configuration at an elevated temperature (55 °C) at 0.1C rate (Fig. S4†). The cell delivered a stable discharge capacity of 78 mA h g^−1^ for 45 cycles with a coulombic efficiency of around 85%. It is interesting to note that the shape of the first charge–discharge curve is similar to the one at room temperature but the coulombic efficiency is lower at high temperatures than that at room temperature, which might be due to the accelerated kinetics during the charging processes at high temperatures. The rate capability of the NCPP–CNT composite from 1C to 20C is presented in Fig. S5.† The discharge capacities of the NCPP–CNT composite at 1C, 2C, 5C, 10C and 20C were 55 mA h g^−1^, 48 mA h g^−1^, 39 mA h g^−1^, 31 mA h g^−1^ and 20 mA h g^−1^, respectively. The charge–discharge curves at different current rates are shown in Fig. S5b.† The NCPP–CNT composite shows excellent cyclability and rate capability at elevated temperatures. The rate capability of the NCPP–CNT composite at room temperature from 1C to 20C is presented in Fig. S6.†

**Fig. 7:**
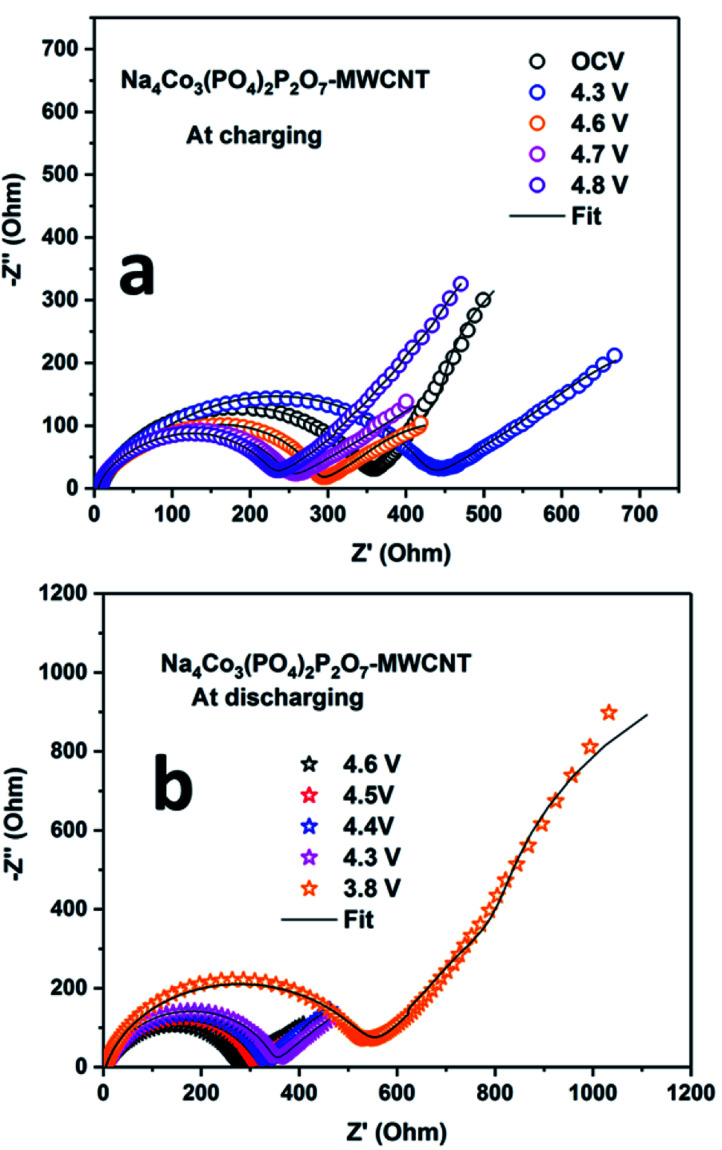
Electrochemical impedance spectra for the Na_4_Co_3_(PO_4_)_2_P_2_O_7_–MWCNT composite electrode during (a) charging and (b) discharging at room temperature.

EIS was carried out during the first cycle at different potentials to determine the solid electrolyte interface (SEI) properties. The Nyquist plots of the NCPP–CNT composite at room temperature and high temperatures are shown in Fig. 7 and S8,† respectively. The scatter points represent the experimental data points and the continuous black line represents the fitted data for the NCPP–CNT composite (Fig. 7 and S8†). The obtained data were analyzed by fitting an electrical equivalent circuit consisting of resistors and constant phase elements (CPE) shown in Fig. S7 (ESI†).^39^ The experimental data were fitted by using the *Z-fit* software. FromFig. 7a, we can infer that during the charging state, the Nyquist plot of the cell at OCV consists of a depressed semi-circle followed by the Warburg element at low frequencies. The depressed semi-circle is observed in a high-frequency range, which is related to the combination of a passivation film on the surface (SF) and the charge transfer process at the interface (CT). The inclined slope observed in the low-frequency range is attributed to the Warburg impedance (*W*_d_), which corresponds to the Na ion diffusion in the bulk of the electrode. While charging up to 4.3 and 4.6 V, only a highly depressed semicircle was observed at high and intermediate frequency ranges and a sloping line was observed in the low-frequency region. After further charging to 4.8 V, the sizes of the high and intermediate frequency semicircles were reduced. In Fig. 7b, we can observe that *R*_CT_ remains unchanged, but the value of *R*_SF_ increases as the voltage is decreased to 3.8 V and the slope of the inclined line at a lower frequency also decreases in the discharge state; this might be due to the mass diffusion processes affected by the formation of large grain boundaries. Additionally, the NCPP–CNT electrode materials with a conducting CNT coating offer good charge transfer kinetics, which provides constant capacity values in long-term cycling.^40,41^ Fig. S9† shows the Nyquist plot for the NCPP–CNT composite electrodes during the (a) charging and (b) discharging states at 55 °C. During discharging, compared to the Nyquist plot obtained at room temperature, the high-temperature Nyquist plots exhibit mostly stable charge transfer resistance (*R*_CT_), which is reflected in the stable discharge capacity. This stability might be due to the formation of highly stable SEI by the reduction of EC and PF_6_^−^ at high voltages. All fitted *R*_E_, *R*_CT_ and *R*_SF_ values during the 1st discharge–charge cycle are listed in Table 1.

**Table 1:** *R*_E_, *R*_CT_ and *R*_SF_ values of the Na/Na_4_Co_3_(PO_4_)_2_P_2_O_7_–MWCNT cell during the 1st discharge–charge cycle at various voltages

		*R*_E_ (Ω)	*R*_SF_ (Ω)	*R*_CT_ (Ω)
Charging	OCV	8.34	47.7	295
	4.3	6.1	62.5	385.2
	4.6	4.5	38.4	225.4
	4.7	4.4	34	195.8
	4.8	4.5	34.4	185.9
Discharging	4.6	4.5	34.6	225.4
	4.5	4.5	36	253.6
	4.4	4.5	36.5	271.3
	4.3	4.5	67.1	268.2
	3.8	4.5	181.2	335.3

**Fig. 8:**
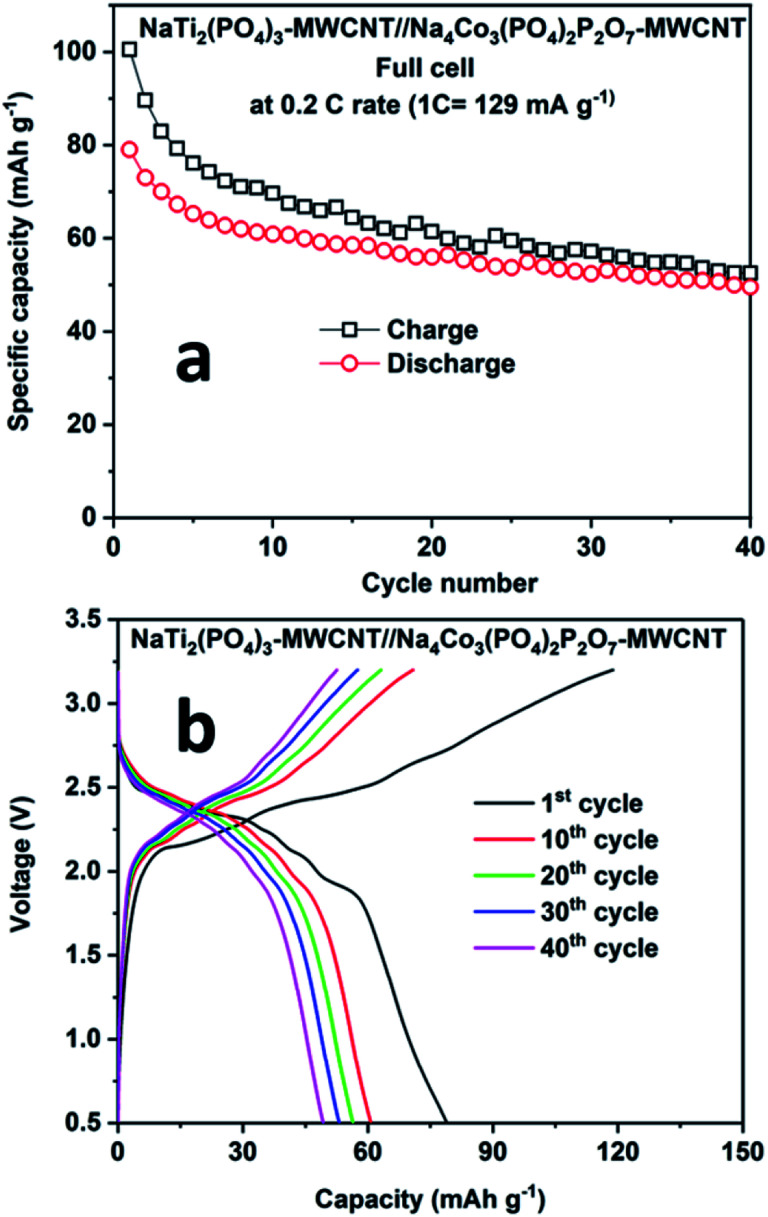
(a) Cyclability results and (b) charge–discharge curves for the NaTi_2_(PO_4_)_3_–MWCNT//Na_4_Co_3_(PO_4_)_2_P_2_O_7_–MWCNT composite at 0.2C rate at room temperature.

Fig. 8 shows the performance of the full cell comprising NaTi_2_(PO_4_)_3_–MWCNT//NCPP–CNT in the electrolyte containing 1 M NaPF_6_ in EC:DMC cycled between 0.5 and 3.25 V at 0.2C rate. The full cell delivered an initial discharge capacity of 78 mA h g^−1^ (current density of 24 mA g^−1^) with a moderate coulombic efficiency of 85% (Fig. 8a). The discharge capacity decreased to 50 mA h g^−1^ after 40 cycles. Fig. 8b shows the charge–discharge voltage profiles for different cycle numbers. The cell comprising NaTi_2_(PO_4_)_3_–MWCNT//NCPP–CNT exhibited an average discharge voltage of around 2.25 V and the typical voltage profile well matched with the voltage difference of NCPP–CNT and NaTi_2_(PO_4_)_3_–MWCNT half-cells.^42^ These results confirm the suitability of the electrolyte containing 1 M NaPF_6_ in EC:DMC for exploring the electrochemical properties of high voltage NCPP–CNT composites.

## 4. Conclusion

We prepared NCPP with MWCNT composite materials by simple sol–gel synthesis followed by calcination at 650 °C in an argon atmosphere. The electrochemical stability windows of the electrolytes containing 1 M NaPF_6_ in EC:DMC and 1 M NaClO_4_ in PC + 5% FEC were explored using LSV curves. The electrolyte containing 1 M NaPF_6_ in EC:DMC showed high voltage stability up to 6.3 V. The cathode composite in the electrolyte containing 1 M NaPF_6_ in EC:DMC delivered stable discharge capacities of 80 mA h g^−1^ and 78 mA h g^−1^ at room and elevated (55 °C) temperatures, respectively. Finally, we demonstrated a full-cell configuration *vs.* NaTi_2_(PO_4_)_3_–MWCNT and it delivered an initial discharge capacity of 78 mA h g^−1^ at 0.2C rate. The NCPP–CNT composite showed excellent cyclability and rate capability at both room and elevated temperatures. This excellent performance might be due to the enhancement in the electronic conductivity of the composite. Hence, the simple sol–gel synthesis of the high voltage NCPP–CNT material with highly stable DMC-based electrolytes can be applied in a wide range of applications in the next-generation rechargeable sodium-ion batteries. The obtained results provide a pathway for improving the electrochemical performance of high voltage cathodes toward the commercialization of SIBs.

## Conflicts of interest

There are no conflicts to declare.

## Supplementary Material

RA-010-D0RA02349C-s001
