# Supplementary material for: Electrochemical studies of a high voltage Na4Co3(PO4)2P2O7–MWCNT composite through a selected stable electrolyte
Source: RSC Adv. 2020 Apr 22;10(27):15983–9. doi: 10.1039/d0ra02349c (PMC9052416; doi:10.1039/d0ra02349c)
Supplement: RA-010-D0RA02349C-s001 [file RA-010-D0RA02349C-s001.pdf]

Electronic Supplementary Material (ESI) for RSC Advances.
This journal is © The Royal Society of Chemistry 2020

Electronic Supplementary Information

Electrochemical studies of high voltage $Na_4Co_3(PO_4)_2P_2O_7$-MWCNT composite through selected stable electrolyte

P. Ramesh Kumar[a], R. Essehli[b] H. B. Yahia[a], R. Amin[b*], I. Belharouak[b]

[a] *Qatar Environment and Energy Research Institute (QEERI), Hamad Bin Khalifa University, Qatar Foundation, 34110, Doha, Qatar.*

[b] *Energy and Transportation Science Division, Oak Ridge National Laboratory, Oak Ridge, TN, USA.*

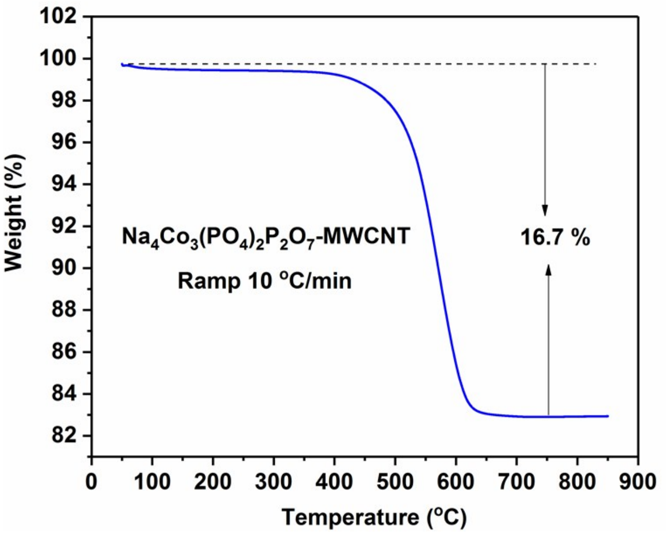

Figure S1. Thermal gravimetric analysis curve for the $Na_4Co_3(PO_4)_2P_2O_7$-MWCNT composite.

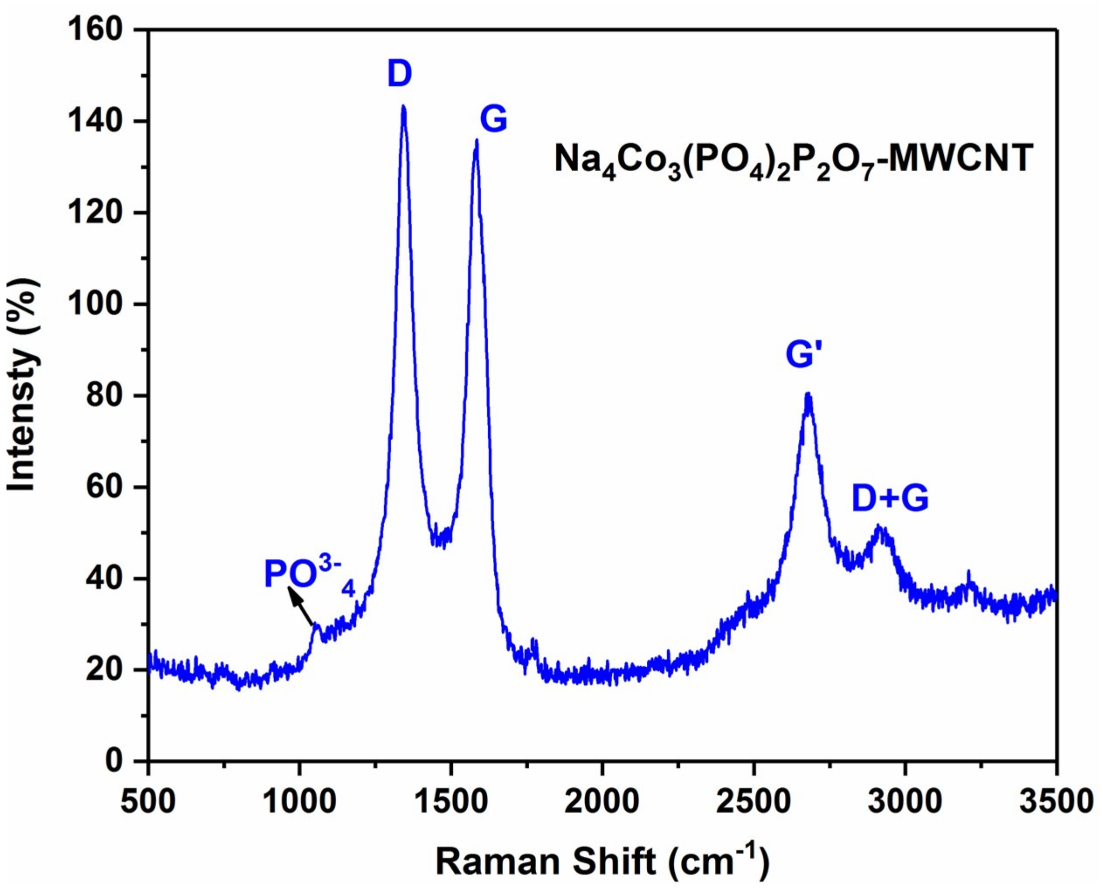

Figure S2. Raman spectra for the $Na_4Co_3(PO_4)_2P_2O_7$-MWCNT composite.

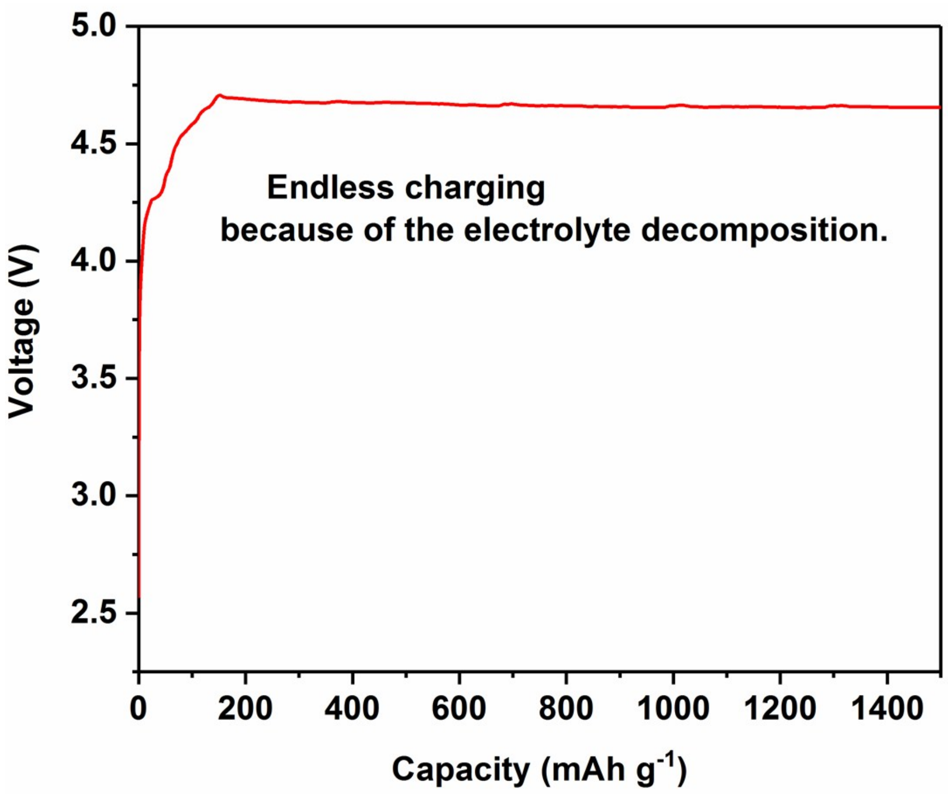

Figure S3. First charging curve for the $Na_4Co_3(PO_4)_2P_2O_7$-MWCNT composite in 1M $NaClO_4$ in PC+ 5% FEC electrolyte.

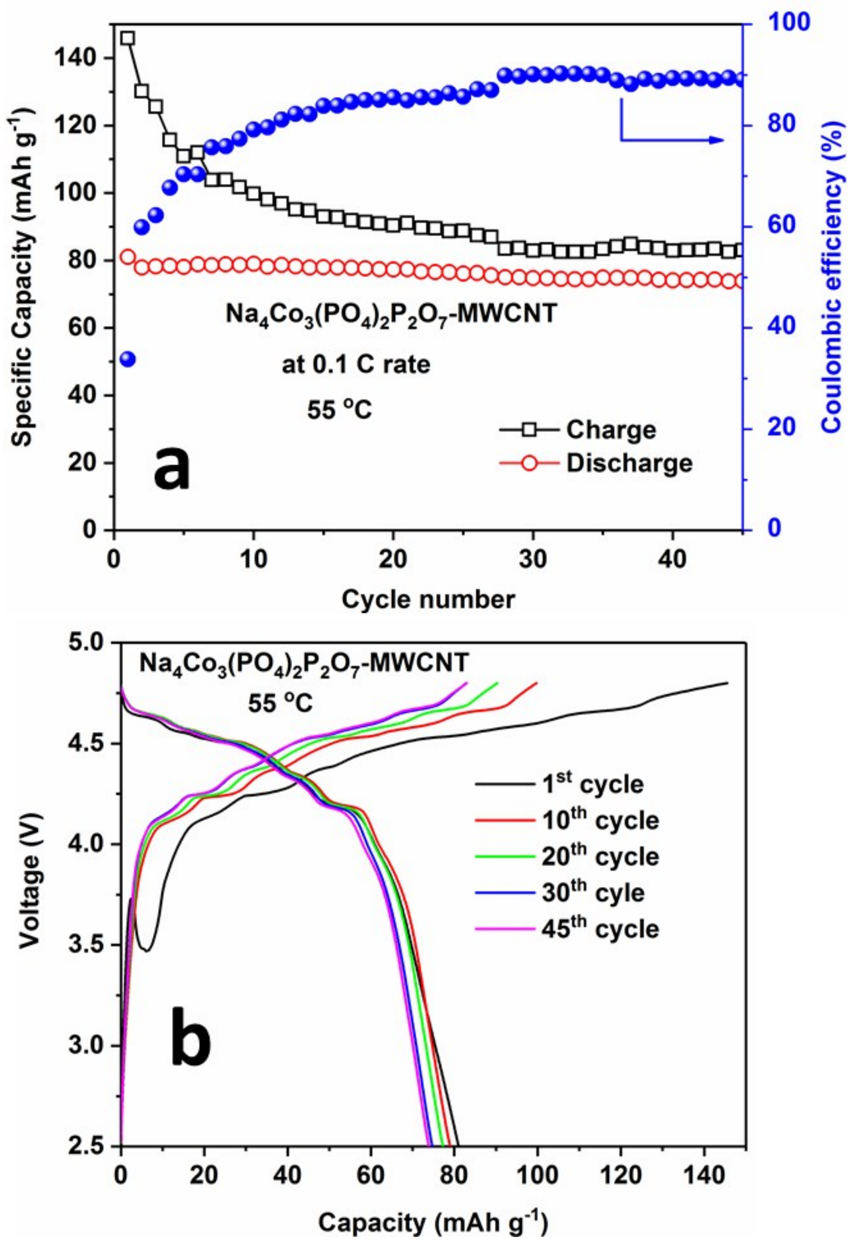

Figure S4. a) Cycleability results and b) Charge –discharge curves for the $Na_4Co_3(PO_4)_2P_2O_7$-MWCNT composite at 55°C.

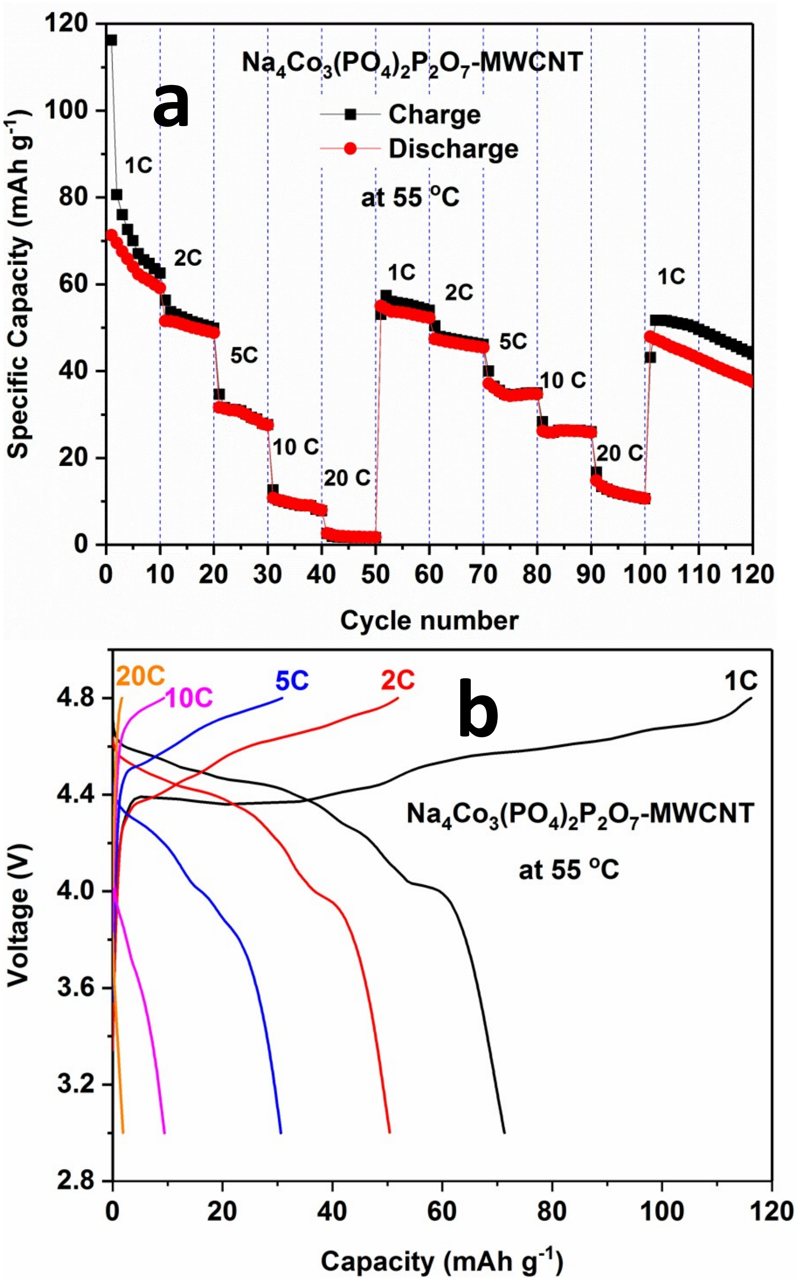

Figure S5. Rate capability results from 1 C to 20 C

for the $Na_4Co_3(PO_4)_2P_2O_7$-MWCNT composite at 55°C.

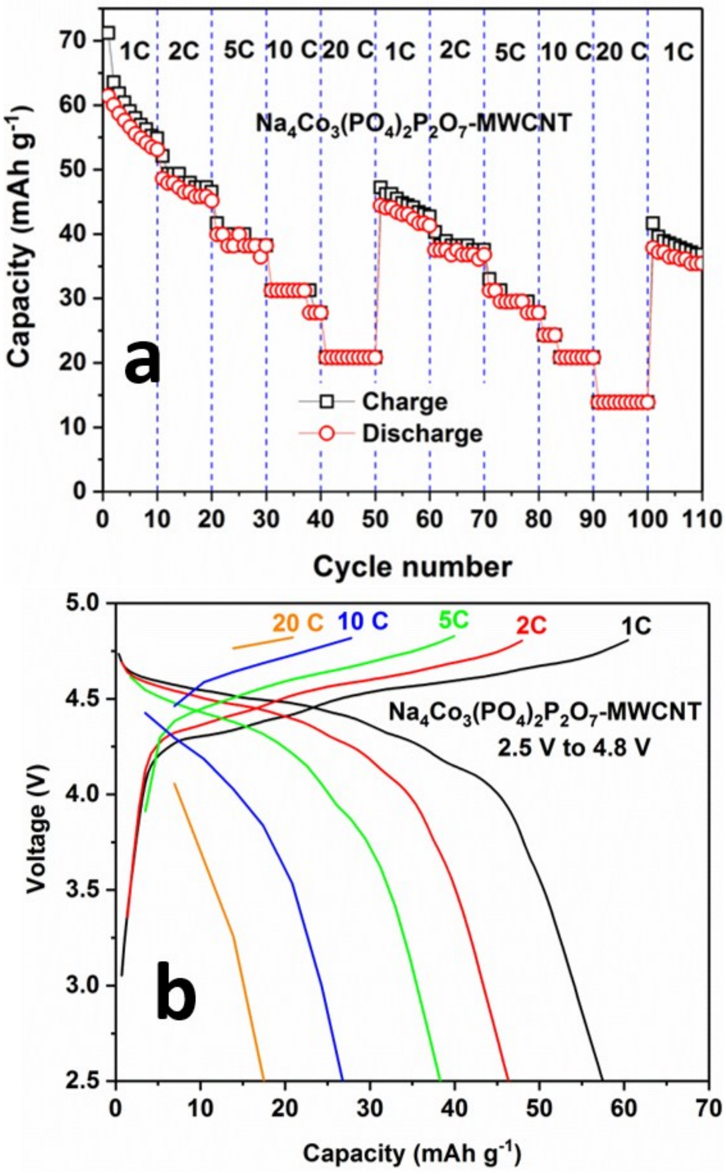

Figure S6. Rate capability results from 1 C to 20 C for the $Na_4Co_3(PO_4)_2P_2O_7$-MWCNT composite at room temperature.

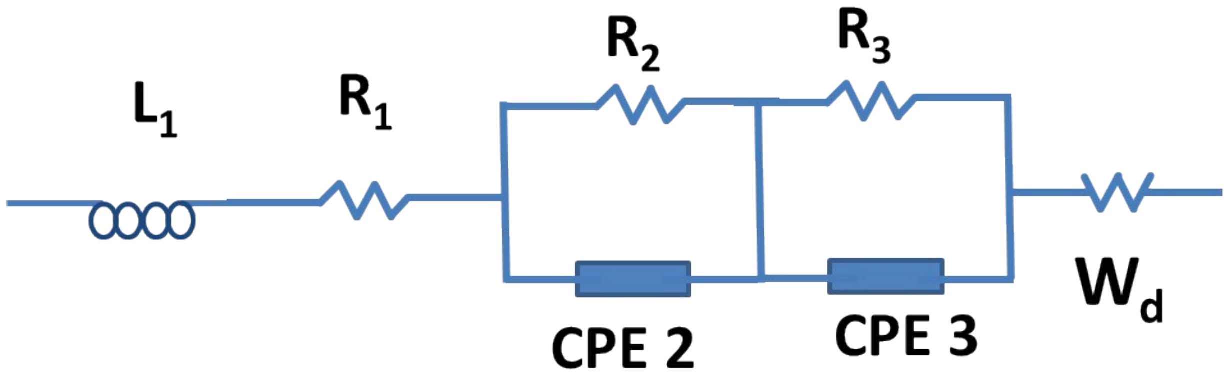

Figure S7: Equivalent electrical circuit model for fitting impedance data.

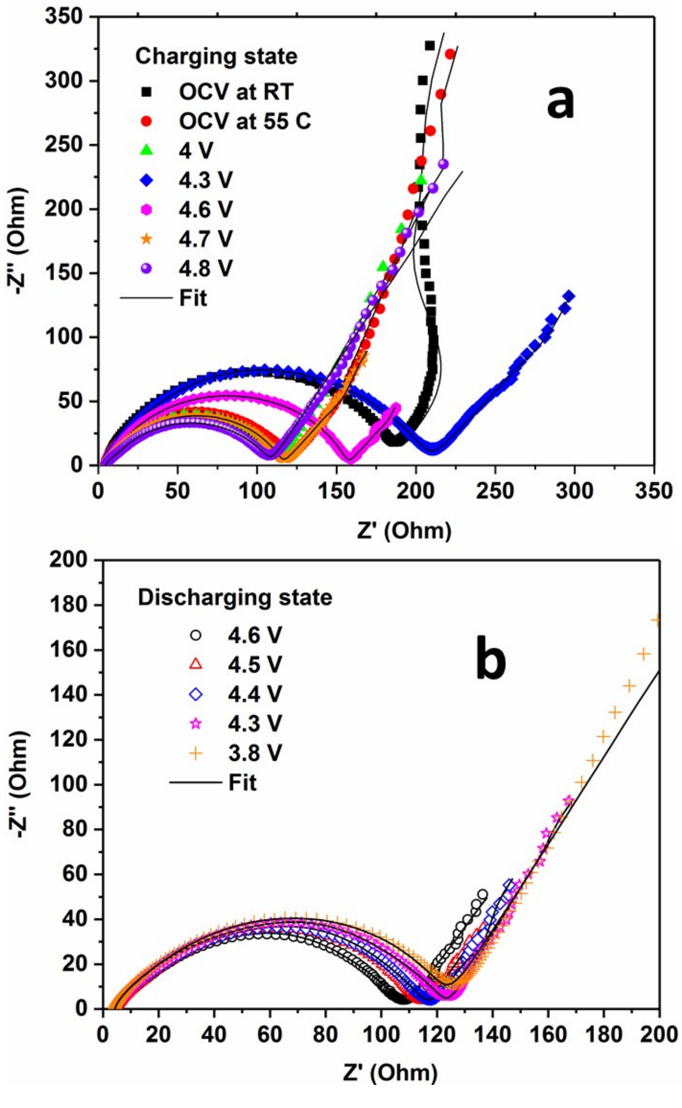
a
Charging state
OCV at RT
OCV at 55 C
4 V
4.3 V
4.6 V
4.7 V
4.8 V
Fit
-Z'' (Ohm)
Z' (Ohm)
b
Discharging state
4.6 V
4.5 V
4.4 V
4.3 V
3.8 V
Fit
-Z'' (Ohm)
Z' (Ohm)

Figure S8. Electrochemical Impedance spectra for the $Na_4Co_3(PO_4)_2P_2O_7$-MWCNT composite electrode during the a) charging and b) discharging states at 55 ^oC.
